# The relative nature of fertilization success: Implications for the study of post-copulatory sexual selection

**DOI:** 10.1186/1471-2148-8-140

**Published:** 2008-05-12

**Authors:** Francisco García-González

**Affiliations:** 1 Centre for Evolutionary Biology, School of Animal Biology (M092), The University of Western Australia, Nedlands, WA 6009, Australia

## Abstract

**Background:**

The determination of genetic variation in sperm competitive ability is fundamental to distinguish between post-copulatory sexual selection models based on good-genes vs compatible genes. The sexy-sperm and the good-sperm hypotheses for the evolution of polyandry require additive (intrinsic) effects of genes influencing sperm competitiveness, whereas the genetic incompatibility hypothesis invokes non-additive genetic effects. A male's sperm competitive ability is typically estimated from his fertilization success, a measure that is dependent on the ability of rival sperm competitors to fertilize the ova. It is well known that fertilization success may be conditional to genotypic interactions among males as well as between males and females. However, the consequences of effects arising from the random sampling of sperm competitors upon the estimation of genetic variance in sperm competitiveness have been overlooked. Here I perform simulations of mating trials performed in the context of sibling analysis to investigate whether the ability to detect additive genetic variance underlying the sperm competitiveness phenotype is hindered by the relative nature of fertilization success measurements.

**Results:**

Fertilization success values render biased sperm competitive ability values. Furthermore, asymmetries among males in the errors committed when estimating sperm competitive abilities are likely to exist as long as males exhibit variation in sperm competitiveness. Critically, random effects arising from the relative nature of fertilization success lead to an underestimation of underlying additive genetic variance in sperm competitive ability.

**Conclusion:**

The results show that, regardless of the existence of genotypic interactions affecting the output of sperm competition, fertilization success is not a perfect predictor of sperm competitive ability because of the stochasticity of the background used to obtain fertilization success measures. Random effects need to be considered in the debate over the maintenance of genetic variation in sperm competitiveness, and when testing good-genes and compatible-genes processes as explanations of polyandrous behaviour using repeatability/heritability data in sperm competitive ability. These findings support the notion that the genetic incompatibility hypothesis needs to be treated as an alternative hypothesis, rather than a null hypothesis, in studies that fail to detect intrinsic sire effects on the sperm competitiveness phenotype.

## Background

A wealth of research inspired by Parker's [1] seminal study on sperm competition has provided overwhelming evidence that mating success is not always equivalent to reproductive success [1-4]. Sperm competition, a direct consequence of polyandry (females mating with different partners within a single reproductive episode), can contribute to the maintenance of promiscuity if females accrue benefits from encouraging the overlap of ejaculates at the site of fertilization. The sexy-sperm hypothesis, a post-copulatory analogue of the sexy-sons model for the evolution of female preferences and male attractiveness [5-7], suggests that polyandrous females could enhance their fitness if fertilization success is genetically correlated to female mating frequency. The good-sperm hypothesis, an analogue of the pre-copulatory good-genes model for mate choice, also suggests that females will accrue genetic quality for their offspring through facilitation of sperm competition, in this case if males with higher sperm competitive ability sire offspring with higher viability [8,9]. Importantly, both the sexy-sperm and the good-sperm model are based on additive (intrinsic) effects of genes influencing a male's sperm competitiveness [10,11]. On the contrary, polyandrous females have also been suggested to benefit from processes based on non-additive genetic effects if multiple mating served as a defence against genetic incompatibilities between them and their partners. The genetic incompatibility hypothesis [12-15] was originally proposed to account for benefits of polyandry in the form of enhanced viability of offspring (i.e., through processes occurring after fertilization). However, avoidance of genetic incompatibility at the pre-fertilization stage, through sperm selection mechanisms based on, for instance, gamete recognition, can also play a role in determining fertilization output [16-18].

In the context of selection based on good-genes, sperm competitive ability is defined as the investment in traits that influence a male's ability to win fertilizations in conditions of sperm competition, under the assumption that males of higher genetic quality can allocate more resources to traits involved in sperm competition [9]. An increasing body of research focuses on investigations of the transitivity or repeatability of fertilization success, or the genetic architecture of traits influencing fertilization success [see reviews in [11,19]]. Support for the notion that sperm competitive ability can be an intrinsic trait comes from different sources. These include studies looking at the repeatability or heritability of fertilization success or paternity success scores [20-27], or examining phenotypic traits thought to be important in sperm competition [11,19,28], but also selection experiments [29] and analysis of the condition-dependence of these traits [28,30-32]. However, current views agree that some factors limit the evolution of sperm competition traits via good-genes processes and contribute to the maintenance of genetic variation among males in traits determining the outcome of sperm competition. These factors include male × female interactions [e. g., [26,30,33-36]], antagonistic pleiotropy [37], sex-biased inheritance [38-41], and ejaculate × ejaculate interactions [34,42,43].

Accurate estimates of sperm competitiveness values for individual males are thus critical for the study of post-copulatory sexual selection, and in particular, to (1) investigate the genetic basis of sperm competitive ability, (2) distinguish between competing hypotheses based on additive or non-additive genetic effects, and (3) further test predictions from these hypotheses. For instance, testing the good-sperm model in phenotypic studies relies on the correlation between a male's sperm competitive ability and his offspring viability [9,44]. However, a pervasive problem that has received little attention arises from the fact that a male's sperm competitiveness is an absolute measure that has to be estimated from a relative measure: fertilization success. Sperm competitive ability of a given male can only be holistically assessed by looking at the output of fertilization trials involving other males in the population acting as sperm competitors. In other words, a male's fertilization success depends not only on his sperm competitive ability but also on the ability of the rival males to win fertilizations.

This study asks for the first time whether fertilization success measures are good estimators of individual sperm competitiveness values. By simulating sperm competition experiments involving pairs of males extracted from a large population characterized by a given distribution of sperm competitiveness I first show that fertilization success is not a perfect predictor of sperm competitiveness. The study further examines the consequences of random effects arising from the relative nature of fertilization success for studies of post-copulatory sexual selection. Simulations of mating trials performed in the context of sibling analysis indicate that the heritability of sperm competitive ability is dramatically underestimated when fertilization success values are used. I discuss the implications of these results for investigations on the genetic nature and the maintenance of genetic variance in sperm competitiveness, and for the study of the evolution of polyandry.

## Methods

### General methods

The analyses consist of three steps. First, simulations of sperm competition experiments involving pairs of males extracted from a large population characterized by a given distribution of sperm competitiveness. Second, the assessment of the efficacy of fertilization success as a predictor of sperm competitive ability. Third, the assessment of the consequences of the deviations between individual fertilization success values and sperm competittive ability values.

Sperm competitiveness is influenced by a number of traits that may include ejaculate volume [45], sperm quality [46,47], sperm morphology [30,48], seminal fluid products [49-51] and genital morphology [24,52], among others. For simplicity, here I will refer to sperm competitive ability as a male's investment in ejaculate volume (numbers of sperm inseminated), which will be assumed to be the primary determinant of fertilization success in a hypothetical species. Nevertheless, this simplification imposes no limitation to the interpretation of the results. The analyses involve the calculation of fertilization success for the second male to mate a doubly mated female. Fertilization success for this arbitrarily chosen male is defined as the proportion of ova that he fertilizes and is denoted F_2_. In practice, fertilization success is generally estimated from P_2_, the observed proportion of offspring sired by the second male calculated at birth or hatching [53]. Here the denomination F_2 _is preferred over P_2 _to emphasize that the analyses focus on "true" fertilization success, i.e., estimated just at conception. The congruence between F_2 _and P_2 _will depend to a large extent on the subsequent viability of offspring between fertilization and paternity assessment [54-56].

Two examples of distributions for sperm competitive ability are used: *S *and *Snormal*, both containing 60000 values of sperm competitiveness in a scale from 0 to 1. Mean sperm competitiveness for the bell-shaped *S *distribution is 0.495 (SD = 0.19, range 0.192–0.999; further details of this distribution can be found elsewhere [56] and in the Additional file 1). Although the distribution *S *exhibits to some extent a shape similar to that of a normal distribution, it deviates significantly from normality (K-S d = 0.08, p < 0.01 ; Lilliefors p < 0.01). A second distribution of values for sperm competitive ability (*Snormal*) uses a normal distribution (x = 0.5, SD = 0.12, Min. = 0.013, Max. = 0.994).

### Fertilization success as a predictor of sperm competitive ability

I have simulated sperm competition trials involving pairing of males from the hypothetical distribution of *S*, or *Snormal*, to calculate fertilization success (F_2_) values assuming a sperm mixing mechanism of sperm competition following the fair raffle principle of Parker [57]. Under the fair raffle, the fertilization success of a male is a function of his investment in sperm competition relative to the investment of the competitor male, and thus F_2 _= *s*_2_/(*s*_1_+*s*_2_), where *s*_1 _and *s*_2 _are sperm competitiveness for the first and second male, respectively, on a scale from 0–1 (without including zero). I have calculated F_2 _values using values of *s*_1 _taken at random from the distribution of sperm competitive abilities and fixed *s*_2 _values of 0.2, 0.3, 0.4, ..., 1 (0.192 is the minimum sperm competitiveness value for the distribution *S*), or 0.02, 0.3, 0.4, ..., 1 (0.013 is the minimum sperm competitiveness value for the distribution *Snormal*). I have simulated 5000 double matings for each category of *s*_2_. Subsequently, from the array of F_2 _values obtained for each fixed category of *s*_2 _I have calculated the probability that F_2 _deviates from *s*_2 _in such a way that the absolute difference between F_2 _and *s*_2 _is higher than 0.1, 0.2 or 0.3.

Differences in the variance between a set of F_2 _values and the *s*_2 _values from which they originate were explored in a second set of simulations. Here I simulated 10000 sperm competition experiments (n = 50 double matings each experiment) by random resampling of *s*_1 _and *s*_2 _values in the distribution of sperm competitiveness *S *or *Snormal*. Subsequently, for each simulated experiment, the coefficients of variation for the set of *s*_2 _values, and for the set of F_2 _values, were calculated. The coefficients of variation were compared using a T-test for dependent samples.

### Consequences of the relative nature of fertilization success on the estimation of genetic variance in sperm competitiveness

Results arising from the analyses above suggested that the relative nature of F_2 _calculations may have important consequences for investigations of the genetic variance in sperm competitive ability. These consequences have been explored with an approach that calculates the intraclass correlation coefficient following the simulation of hypothetical sibling analyses. The genetic design simulated involves screening of the fertilization success of 8 offspring for each of 50 sires. Sperm competitive ability for each sire (*s*_2-*Sire*_) has been assigned by random extraction of sperm competitive ability values from the distribution *S*. The values of sperm competitive ability for each individual offspring of each sire have been calculated as *s*_2-*Offspring *_= (*s*_2-*Sire *_+ *R*)/2, where *R *is a random number between 0 and 1 from a uniform distribution. This protocol simulates heritable variation for sperm competitive ability, and results in moderate random deviations of *s*_2-*Offspring *_from *s*_2-*Sire*_. For each experiment (sibling analysis involving 50 sires), a one-way Anova is carried out to obtain the variance components (among sires, and within sire), which allows the calculation of the intraclass correlation coefficient. The intraclass correlation coefficient, *t*, is defined as the phenotypic correlation between sibs [58], which provides an estimate of the fraction of the phenotypic variance attributable to differences among sires. The simulated set up can be taken as a paternal half-sib design in which a single offspring from each of 8 unrelated females mated to a male is assayed [58], or a full sib design in which each sire is mated to only one female, and 8 offspring per female are assayed. In the case of the paternal half-sib analysis, heritability, *h*^2 ^= 4*t*, under the assumption of negligible epistasis and common environmental effects, whereas in the case of the full sib analysis, *h*^2 ^= 2*t*, provided that there were no dominance effects and no common environmental effects [58].

The key point of the analysis performed is that the protocol allows the calculation of the "true" intraclass correlation coefficient for the trait "sperm competitive ability" (i.e., *s*_2_). To investigate the influence of the relative nature of F_2 _measures on estimates of the additive genetic variance of sperm competitive ability this "true" *t *is compared to the *t *that would be obtained in empirical studies; i. e., inferring genetic additive variance in sperm competitive ability from fertilization success values (F_2_). Therefore, the values of sperm competitiveness for the offspring in the hypothetical design have been put in the context of fertilization success values following double matings. I have calculated fertilization success values for the offspring (F_2-*Offspring*_) using each offspring's *s*_2-*Offspring *_value (generated from the *s*_2-*Sire*_) and random-extracted *s*_1 _values from the distribution *S*. Subsequently, in the same way that *t *was calculated using "real" sperm competitiveness values, I have calculated the intraclass correlation coefficient using the F_2-*Offspring *_values derived from the *s*_2-*Offspring*_. The difference between the intraclass correlation coefficient for *s*_2-*Offspring *_and for F_2-*Offspring *_would therefore inform on how the estimations of additive genetic variance in sperm competitive ability are affected by the relative nature of fertilization success values. The whole protocol has been replicated 10000 times, each time following a new random extraction of sperm competitive ability values for the sires. The intraclass correlation coefficients using *s*_2 _data and their associated intraclass correlation coefficients using F_2 _data have been compared using a T-test for dependent samples. The same methodology has been used using sperm competitive ability values from the *Snormal *distribution. Simulations have been carried out using PopTools 2.7.5 [59], while Statistica 6.0 [60] has been used for data analyses.

## Results

### Fertilization success as a predictor of sperm competitive ability

**Figure 1:**
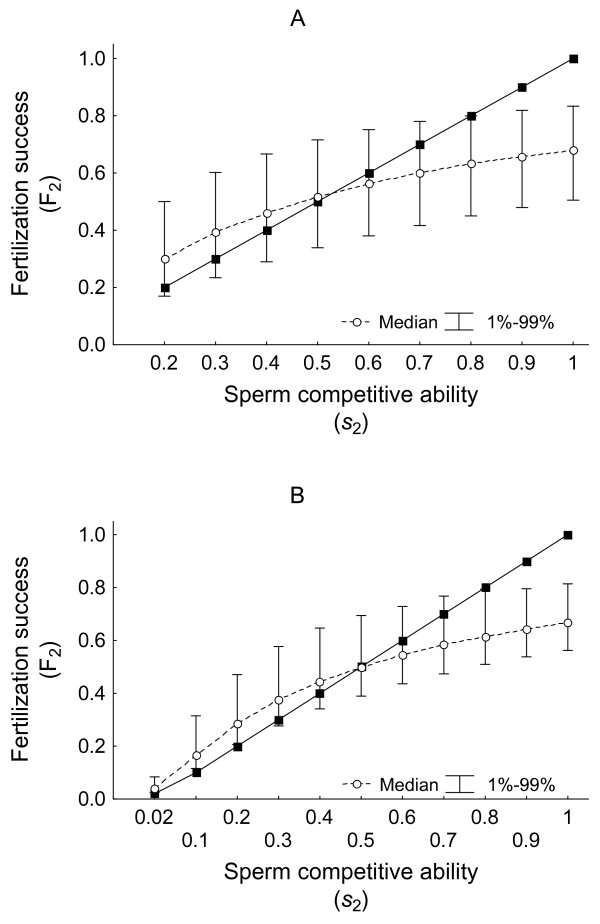
**Variation in fertilization success values**. The figure represents the variation in fertilization success values for the second male (open circles and whiskers) that can be originated from the same sperm competitive ability value for the second male. The example uses fixed values of *s_2_*, and *s_1_*values extracted at random from the distribution of sperm competitiveness *S *(A) or *S normal *(B). If F_2 _were a perfect estimator of sperm competitive ability the solid line should be obtained.

Every single value of sperm competitive ability can generate a wide range of fertilization success values as a result of variation in the sperm competitive ability of rival males (Figs. 1A and 1B). The probabilities of mistakenly inferring sperm competitive ability values can be high (see Additional file 2). The probability curves reflect assymetries in the degree and direction of biases, which depend on the sperm competitive ability value being tested and the influence of values that are overrepresented in the population (e.g., average sperm competitiveness values in distributions suggestive of stabilizing selection on sperm competitive ability)(see Additional file 2).

An important consequence of the patterns of bias is that the variance for the observed ability of males to win fertilizations is reduced. This is confirmed in the analysis in which the coefficients of variation for *s*_2 _values and the associated F_2 _values are calculated following the simulation of sperm competition experiments. When sperm competitive ability values are randomly extracted from the distribution *S*, the mean (± SD) coefficient of variation for the set of *s*_2 _values is 38.1% ± 3.5 (n = 10000 experiments of sample size 50 each) compared to 25.7% ± 2.4 for the set of associated F_2 _values (T-test for dependent samples t_9999 _= 391.6, p <<0.0001). A lower but still highly significant decrease in the coefficient of variation is found when sperm competitive ability values are extracted from the *Snormal *distribution: 23.8% ± 2.5 compared to 17.7% ± 2.0, for *s*_2 _values and associated F_2 _values respectively (T-test for dependent samples t_9999 _= 286.9, p <<0.0001).

### The relative nature of fertilization success and the estimation of genetic variance in sperm competitiveness

**Figure 2:**
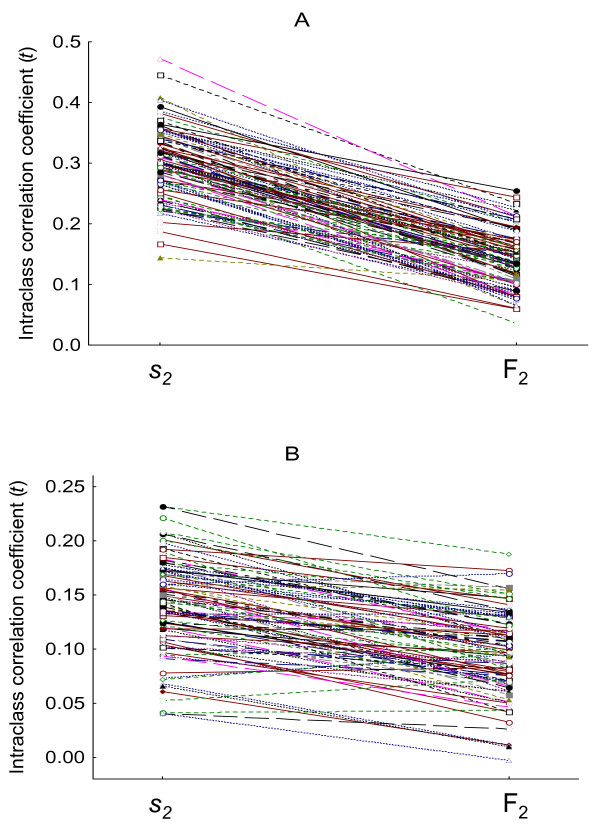
**Reduction in the intraclass correlation coefficient due to variance arising from the relative nature of fertilization success**. The figure shows the intraclass correlation coefficient for sperm competitive ability (*s*_2_) and the associated coefficient when sperm competitive ability is estimated using fertilization success values (F_2_). Each line pertains to a single sib analysis (involving 50 sires and 8 offspring per sire). Only a random subsample of 100 sib analyses is shown. Sperm competitive ability values were extracted from the distribution *S *(A) or *Snormal *(B).

The consequences of the relative nature of F_2 _values for investigations of genetic variance in sperm competitive ability have been examined with analyses of the intraclass correlation coefficient (*t*), recreating hypothetical studies using fertilization success values as proxy of sperm competitive ability (see Methods), such as it is done in real experiments. Results from these analyses show that the heritability of sperm competitive ability, as inferred from fertilization success, can be seriously underestimated. The comparison between *t *generated from "true" sperm competitive ability values and that generated from the F_2 _values arising from those sperm competitive ability values shows that *t *is decreased to a great extent using F_2 _values. When sperm competitive ability values are randomly extracted from the distribution S, the mean (± SD) *t *for sperm competitive ability (*s*_2_) calculated from 10000 experimental genetic designs (50 sires and 8 offspring per sire in each experiment) is 0.30 ± 0.06 compared to 0.14 ± 0.04 when using the set of associated F_2 _values (T-test for dependent samples t_9999 _= 366.4, p <<0.0001) (See Fig. 2A). This means an average reduction for *t *of around 50%. For example, under the conditions described in the methods, heritabilities of sperm competitiveness calculated in full-sib experiments of around 0.6 will be on average estimated as being 0.3 because of the deviations that fertilization success values impose on the real sperm competitive ability values. Similarly, a significant decrease in the intraclass correlation coefficient is obtained when sperm competitive values are extracted from the distribution *Snormal*: 0.15 ± 0.05 compared to 0.10 ± 0.04, for *s*_2 _values and associated F_2 _values respectively (T-test for dependent samples t_9999 _= 162.5, p <<0.0001) (Fig. 2B).

## Discussion

Research on sperm competition has increased exponentially since the first formal account of its implications [1] and it is now recognized as a powerful force shaping numerous traits in males and females [2], including polyandrous behaviour [5]. In many cases the understanding of post-copulatory processes relies on the measurement of one critical parameter: sperm competitive ability of individual males. However, accurately determining sperm competitive ability presents some problems, partially because it involves third parties. This study cautions that fertilization success is not a perfect predictor of sperm competitive ability, because of the relative nature of the calculation of fertilization success measures. Fertilization success values render biased sperm competitive ability values. Importantly, this bias is not constant along all the range of sperm competitiveness values and a consequence of the patterns of bias is that variance for the observed ability of males to win fertilizations is significantly reduced. Critically, as shown in simulations of sibling analyses, the heritability of sperm competitive ability is seriously underestimated when fertilization success (or paternity success) values are used. Thus, the ability to detect additive genetic variance underlying the sperm competitiveness phenotype is hindered.

A number of factors, including interactions between male and female genotypes [36,43], have been suggested to contribute towards the maintenance of genetic variation in sperm competitive ability or in traits determining sperm competitive ability [reviewed in [11,19,61]]. The existence of male × male interactions may further complicate the determination of the unit of selection for sperm competition [34,43]. However, the role of male × male interactions in maintaining genetic variation is still uncertain. Recently, Bjork et al [42] have carried out a thorough examination of ejaculate × female and ejaculate × ejaculate interactions on sperm offense and defence in *Drosophila melanogaster *using a complex experimental set up that allowed them to measure the repeatabilities of sperm precedence (estimated from paternity success values) across multiple sperm competition trials. Bjork et al [42] found that both sperm offense and defense were highly repeatable in matings involving the same pair of males and the same female. These repeatabilities declined when the same pair of males was mated to different females, indicating that the outcome of sperm competition responds to interactions between male × female genotypes. Finally, the repeatabilities of paternity scores disappeared when males competed each time against different rival males within different females. It has been suggested that this kind of result supports the notion that the maintenance of non-heritable variation in sperm competitive ability is due to interactions between competing males [34,42]. It is important to know, however, whether scores showing low consistency when rival males are changed are due to non-additive variance arising from genotypic male by male interactions or to random, purely environmental, effects due to the relative nature of fertilization success (or paternity success) measures. Ejaculate × ejaculate interactions can be due to genotypic interactions *sensu stricto *between competing males (i.e., due to polymorphism in genes responsible for sperm competitiveness), implying non-transitivity in sperm competitive ability. Prout and Bundgaard [62] provide an example of this scenario, in which genotype *AA *outcompetes genotype *Aa*, and *Aa *is superior that *aa*, but *aa *is superior than *AA*. In this case, it would not be possible to rank males on the basis of their sperm competitiveness, and as Clark [34] points out, the success in sperm competition of a particular genotype will depend on the presence and frequencies of other male genotypes in the population. It seems clear that this type of ejaculate × ejaculate interaction would indeed contribute to the maintenance of polymorphism in genes determining sperm competitive ability [34,43]. Alternatively, ejaculate × ejaculate interactions can be observed as the result of the relative nature of fertilization/paternity success calculations, even if sperm competitive ability is an intrinsic trait allowing ranking of males: males will score better or worse depending on the sperm competitive abilities of rival males. In this case, ejaculate × ejaculate interactions would explain variance in the outcome of sperm competition, but it is less clear that these interactions contributed towards the maintenance of genetic variation in sperm competitive ability. The evolutionary consequences of these two types of ejaculate × ejaculate interactions differs, and it seems important to investigate the existence of genetic variation in sperm competitive ability controlling, if possible, for random effects arising from the relative nature of fertilization success. Accounting for these effects would inform on whether sperm competitive ability can be treated as an intrinsic trait or whether it should be treated as interacting phenotype [19,63-65].

As it has been recently pointed out by Dowling et al. [65], studies examining genetic variance in the outcome of sperm competition have generally found lower levels of additive genetic variance than studies focusing on specific traits with a role in sperm competition. Results in this paper highlight that confounding random effects arising from the relative nature of fertilization success could explain this discrepancy. While these effects do not affect estimations of genetic variance in specific sperm competition traits, because in these cases absolute measures are taken (e.g., testis size of individuals is measured), they are likely to confound estimations of genetic variance underlying holistic measures of sperm competitiveness. This, however, does not mean that the study of absolute specific traits should be preferred over the study of fertilization success, or vice versa. Fertilization success is the only integrative measure for the outcome of sperm competition and the foremost predictor of absolute sperm competitive ability in general terms. The investigation of the sources of variation in the observable output of sperm competition is warranted [20,23,24,27,34,42,66], and both types of studies (those focusing on a few traits and those focusing on the sperm competitiveness phenotype) are important if we are to advance the understanding of post-copulatory sexual selection.

Interpretations on the plausibility of good-genes or compatible-genes underlying the genetic benefits of polyandrous behaviour often rely on the detection of additive (intrinsic) effects of genes influencing a male's sperm competitiveness [10,11]. However, obtaining evidence for intrinsic sire effects on the sperm competitiveness phenotype is impeded to some degree due to random effects arising from the background in which sperm competitiveness is estimated. This study, therefore, supports the notion that a lack of evidence for sire effects on fertilization success should be taken with caution, and that the genetic incompatibility hypothesis needs to be treated as an alternative hypothesis, rather than a null hypothesis, when testing for post-copulatory processes based on good genes.

The simulations of sperm competition mating trials demonstrate that there can be significant additive genetic variation in sperm competitive ability that researchers measuring fertilization success may fail to detect. Given that it is fertilization success that ultimately determines the patterns of paternity, it could be argued that females facilitating sperm competition will encounter the same problem as researchers staging random mating trials and that they will not be able to obtain genetic benefits. However, fertilization success is a multiple trait that is mainly determined by the action and interaction of absolute traits contributing to sperm competitiveness, which are the real targets of selection (a useful analogy could be, for instance, mating success, which may be determined by a number of traits such as body size, vigour, size of ornaments, etc.). Importantly, the genetic benefits that a polyandrous female can obtain will be determined by the existence of additive genetic variance in traits conferring sperm competitive ability, because these traits will be inherited by the offspring and they will determine to a great extent their subsequent fertilization success. We can imagine a scenario in which there is one male, *M*, with high absolute sperm competitive ability (0.9 on a scale from 0 to 1), mating with two females: female A, previously mated with another male of high absolute sperm competitive ability (0.85), and female B, previously mated with a male of low sperm competitive ability (0.01). Fertilization success for the male *M *is clearly not repeatable across these matings (fertilization success will be 0.51 when mated to female A, and 0.99 when mating with female B). However, if sperm competitive ability is heritable, females A and B will benefit through the offspring sired by male *M *because of the inheritance of high absolute sperm competitive ability that will confer, on average and despite stochastic influences (contingent on the distribution of sperm competitiveness in the population of males), high fertilization success. It is, therefore, the existence of additive genetic variance in traits contributing to sperm competitive ability that determines the potential for the acquisition of good genes through post-copulatory processes in polyandrous females.

It is important to bear in mind that the confounding effects imposed by the relative nature of fertilization success are particularly severe when sperm competitive ability for the individual is the parameter with relevance for inferring post-copulatory processes. In addition, it is worth mentioning that the magnitude of the random effects would depend on the distribution of sperm competitive ability and on the mechanism of sperm competition. However, these effects are likely to occur whenever there is variation among males in sperm competitive ability and some degree of sperm mixing (including the loaded raffle, [67]), or whenever the outcome of sperm competition is determined by interactions between the determinants of paternity of competing males. Finally, it is important to take into account that fertilization success is generally estimated from paternity success at hatching or birth. In this case, differential embryo viability across competing males may imply another source of variation that would further obscure examinations of additive genetic variance in sperm competitive ability [56].

The fact that individual fertilization success values are not always a reflection of sperm competitive ability would generate confounding effects in analyses based on the ranking of males, in particular those investigating the association between sperm competitiveness and phenotypic or life-history traits. Putting the measures of fertilization success in context with those from rival males can alleviate the problem imposed by its relative nature in these studies. Some other methodological measures could ameliorate the problems imposed by the relative nature of fertilization success. In general terms, random extraction of males from the population together with the use of large sample sizes to calculate fertilization success values are advisable to minimize random effects influencing the degree to which fertilization success does not reflect sperm competitiveness. In studies looking at the genetic variance of sperm competitiveness, several approaches could be adopted, although none of them is probably a perfect solution. Sperm competitive ability could be tested against standardised sperm competitors. The use of either the same male or the same set of tester rival males would minimize the problems imposed by the relative nature of fertilization success. This approach will analyse genetic variance in sperm competitive ability in absence of not only variation due to random effects, but also due to genotypic ejaculate × ejaculate interactions. This approach is, however, difficult to implement in most species with internal fertilization due to confounding age effects or mating history effects. In *D. melanogaster*, a promising line for the study of additive genetic variation is hemiclonal analysis (members of a single hemiclone share a random genomic haplotype) [27]. This methodology presents some important strengths including that it virtually screens the entire genome, and that measures of genetic variation are devoid of maternal effects, dominance, and practically epistatic variation [27]. Using this technique, Friberg et al. [27] recently found low but significant heritable variation in offensive and defensive sperm displacement in *D. melanogaster*. Hemiclones could be tested against standardized competitor males; for instance, members of a single hemiclone could be used as competitors for screening variation in sperm competitive ability among an array of hemiclones. In this way, differences among hemiclones without the influence of random effects due to differences in the background in which sperm competitiveness is assayed could be determined. Several species of external fertilizers or species for which artificial insemination/fertilization techniques are available could also be models to examine sources of variance in fertilization success using a standard background with which to assess sperm competitiveness.

## Conclusion

Sperm competitive ability is typically estimated following fertilization trials involving rival males. Thus, a given value of sperm competitive ability, which represents the absolute investment in traits that convey advantage under conditions of sperm competition, has the potential to generate a wide range of different fertilization success values, depending on the sperm competitive ability of rival males. Here I have shown that the relative nature of the calculation of fertilization success means that the inference of sperm competitive ability is biased. Random effects arising from the way that fertilization success is calculated may confound investigations on the genetic nature and the maintenance of genetic variance in sperm competitiveness, an area that is generating considerable debate [24,25,34,37,38,42,49]. Indeed, results in this study demonstrate that the detection/estimation of additive genetic variance in sperm competitiveness is hampered when using fertilization success. Given that observed low additive genetic variance in fertilization success is often taken as support for post-copulatory processes based on genetic incompatibilities driving polyandrous behaviour [10,11,61], these results have important implications for studies of the evolution of polyandry. The effects shown in this study suggest that taking the genetic incompatibility hypothesis as the null-hypothesis when tests for repeatability/heritability in fertilization success fail to support good-genes processes is not advisable.

## Authors' contributions

FG-G conceived the study, performed the simulations, analyzed the data, and wrote the manuscript.

## Supplementary Material

Additional file 1 *S*, the frequency distribution of sperm competitive ability used as an example. Click here for file

Additional file 2 Probabilities for the deviations between fertilization success and sperm competitive ability. Click here for file
