# Supplementary material for: The relative nature of fertilization success: Implications for the study of post-copulatory sexual selection
Source: BMC Evol Biol. 2008 May 12;8:140. doi: 10.1186/1471-2148-8-140 (PMC2408597; doi:10.1186/1471-2148-8-140)
Supplement: Additional file 1 — S, the frequency distribution of sperm competitive ability used as an example. [file 1471-2148-8-140-S1.pdf]

The relative nature of fertilization success: implications for the study of post-copulatory sexual selection - Francisco García-González

ADDITIONAL FILE 1

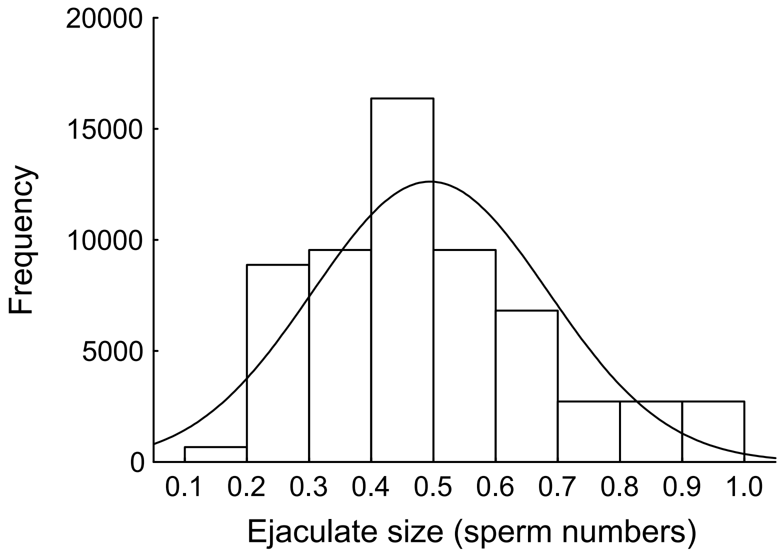

Supplementary Figure 1:
***S*, the frequency distribution of sperm competitive ability used as an example**

Mean ejaculate size (sperm competitiveness), in a scale from 0 to 1, is 0.495 (SD = 0.19, range 0.192-0.999) (the line indicates the normal curve). This distribution is based on a distribution of numbers of sperm inseminated across males that has been taken from the literature, and it has been used elsewhere [1]. Briefly, numbers of sperm stored in the spermatheca after single matings for the fly *Bactrocera* (*Dacus) cucurbitae* have been taken from Yamagishi and Tsubaki [2] (x = 6211, SD = 2439, Min. = 2400, Max. = 12500, n = 44), using their Figure 1 data for copulations longer than 4 hours (i.e., after complete sperm transfer). I adjusted variation of the real sperm number values (sperm competitiveness) to a scale of 0 to 1 by dividing each score by the highest score, and then generated an identical distribution of sperm competitive ability but containing a larger number of values. By grouping the real sperm number values in the categories 0-0.1, 0.1-0.2,...,0.9-0.1, and calculating the frequency of each category, I have created continuity in sperm competitiveness by generating random numbers that adjust to the frequency of each category. The result is a distribution of values for sperm competitive ability (henceforth *S*) identical to the real distribution of sperm numbers (same shape, mean, range, and coefficient of variation) but containing 60000 different values. The distribution of sperm inseminated in *B. cucurbitae* is merely used as a biologically realistic example of the distribution of sperm competitive ability in a population of males. The real sperm competition mechanism in *B. cucurbitae* is not relevant in the context of the present paper, but nonetheless ejaculate size in this species determines paternity when females mate with multiple males in rapid succession, and the mechanism of sperm competition seems to be one of sperm mixing [3], the mechanism simulated in this study.

1. García-González F: **Male genetic quality and the inequality between paternity success and fertilization success: consequences for studies of sperm competition and the evolution of polyandry**. *Evolution* 2008, **in press**:doi:10.1111/j.1558-5646.2008.00362.x.
2. Yamagishi M, Tsubaki Y: **Copulation duration and sperm transfer in the melon fly, *Dacus cucurbitae* Coquillett (Diptera, Tephritidae)**. *Appl Entomol Zool* 1990, **25**(4):517-519.
3. Yamagishi M, Itô Y, Tsubaki Y: **Sperm competition in the melon fly, *Bactrocera cucurbitae* (Diptera: Tephritidae): effects of sperm longevity on sperm precedence**. *J Insect Behav* 1992, **5**:599-608.
