# Supplementary material for: The relative nature of fertilization success: Implications for the study of post-copulatory sexual selection
Source: BMC Evol Biol. 2008 May 12;8:140. doi: 10.1186/1471-2148-8-140 (PMC2408597; doi:10.1186/1471-2148-8-140)

The relative nature of fertilization success: implications for the study of post-copulatory sexual selection - Francisco García-González

ADDITIONAL FILE 2

Probabilities for the deviations between fertilization success and sperm competitive ability

To assess the potential for confounding effects arising from the disparity between fertilization success and sperm competitiveness, I have calculated the probability of obtaining F_2 values such that the absolute difference between F_2 and the s_2 value from which F_2 arises is higher than 0.1, 0.2 or 0.3 (e. g., for a s_2 value of 0.42, a probability for an absolute difference >0.2 means the probability that F_2 values arising from this value are higher than 0.62 or lower than 0.22). The probabilities of mistakenly inferring sperm competitive ability values can be high (see below Supplementary Figures 2A and 2B). For instance, when working with a distribution of sperm competitiveness such as *S*, the probability of obtaining absolute F_2- s_2 deviations lying within 0.2-0.3 is higher than 0.05 for almost all the range of sperm competitiveness values (excluding intermediate s_2 values). Importantly, high values of sperm competitive ability are underestimated to a great extent (Supplementary Figure 2A). For example, there is a 70% chance that a s_2 value of 0.9 is estimated as being lower than 0.7, and it will be estimated as being lower than 0.6 with a probability equal to 0.25 (the lowest value that a $s_2 = 0.9$ can take is 0.473). When the sperm competitive abilities of the males in the population exhibit a normal distribution (*Snormal*) the same patterns arise, and although the probabilities for high deviations between F_2 and s_2 are lower, the probabilities of underestimating high values of sperm competitive ability are considerable (Supplementary Figure 2B).

The probability curves in Supplementary Figure 2 reveal interesting patterns. For the distributions of sperm competitive ability taken as examples, most of the males in the population exhibit medium values, as is likely to happen in nature if sperm competitive ability is being under stabilizing selection. If males are paired at random, the resulting fertilization success values would be highly influenced by these medium values, with the consequence that there will be a tendency to underestimate and overestimate high and low values of sperm competitive ability, respectively. It would be less likely that a male with very high s_2 is competed against a male with very low or very high sperm competitiveness than with a male of medium sperm competitiveness. This would result in biased F_2 values towards the average sperm competitiveness value (e.g., most of the F_2 values rendered by males with $s_2 = 0.9$ would be around $0.9/(0.5+0.9) = 0.64$, if 0.5 is the mean of the distribution of sperm competitiveness). The same applies for moderately low s_2 values, which would also result in a bias towards the average sperm competitiveness values for the population. However, such bias would occur to a lesser extent because of the comparatively lower weight of low values of sperm competitive ability in a fertilization trial (e.g., most of the F_2 values rendered by $s_2 = 0.1$ would be around $0.1/(0.5+0.1) = 0.17$). Indeed, the bias is negligible for very low values of sperm competitiveness (see Supplementary Figure 2B, and note the difference between the shape of the probability curves for the distribution *Snormal* and *S*, which responds to the fact that the distribution *S* does not contain very low values of sperm competitiveness). Importantly, large biases for the estimations of sperm competitive ability are absent when testing medium s_2 values.

Supplementary Figure 2: Probabilities for the deviations between fertilization success and sperm competitive ability

The figure shows the probabilities that a given value for the sperm competitive ability of the second male (s_2) generates a fertilization value for that male (F_2) such that the absolute difference between F_2 and s_2 is >0.1 (solid circles), >0.2 (open squares) or >0.3 (open circles). Second males are paired with sperm competitors taken at random from the distribution *S* (A) or *Snormal* (B). For each category of s_2, the probabilities have been extracted from 5000 double mating trials (see Methods). The horizontal line indicates a probability of 0.05.

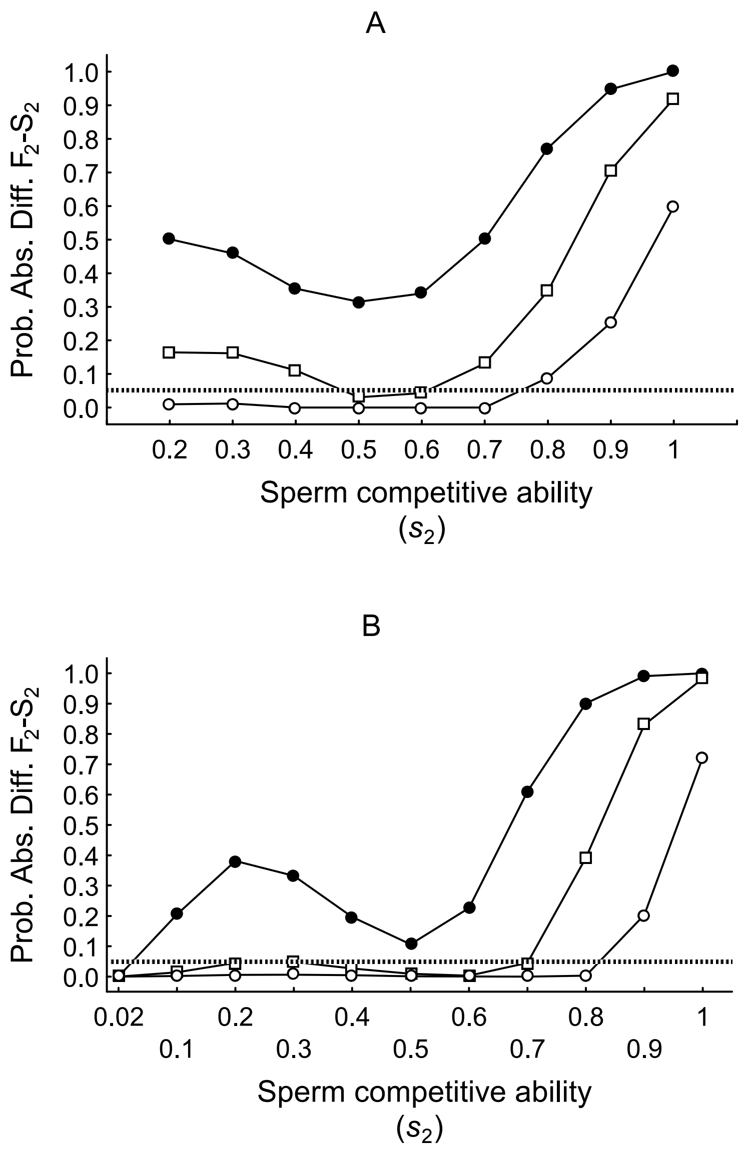

Supplement: Additional file 2 — Probabilities for the deviations between fertilization success and sperm competitive ability. [file 1471-2148-8-140-S2.pdf]
